# Lignin-Derived Ionic Hydrogels for Thermoelectric Energy Harvesting

**DOI:** 10.1021/acsapm.4c03816

**Published:** 2025-03-03

**Authors:** Nicolás Menéndez, Muhammad Muddasar, Mohammad Ali Nasiri, Andrés Cantarero, Clara M. Gómez, Rafael Muñoz-Espí, Maurice N. Collins, Mario Culebras

**Affiliations:** † Institute of Materials Science (ICMUV), 16781 University of Valencia, C/ Catedràtic José Beltrán 2, 46980 Paterna, Spain; ‡ Stokes Laboratories, School of Engineering, Bernal Institute, 8808 University of Limerick, Limerick V94 T9PX, Ireland; § Institute of Molecular Science (ICMol), University of Valencia, C/ Catedràtic José Beltrán 2, 46980 Paterna, Spain; ∥ SFI Centre for Advanced Materials and BioEngineering Research, Dublin D02 PN40, Ireland

**Keywords:** lignin, thermoelectric materials, ionic conductor, hydrogels, sustainable

## Abstract

Thermoelectric materials are attracting attention for their ability to convert heat into electricity, traditionally assessed through a figure of merit (*ZT*) depending on the electrical conductivity, Seebeck coefficient, and thermal conductivity. Developing efficient ionic thermoelectric materials presents challenges as they cannot integrate directly into standard generators. However, they can utilize the ionic thermoelectric effect to charge supercapacitors. This study investigates lignin, an abundant plant-based waste, as a basis for ionic thermoelectric systems, combining sustainability and thermoelectric efficiently. Lignin-based hydrogels with varying compositions were examined for their thermoelectric properties, revealing gigantic ionic Seebeck coefficients of up to 30.4 mV K^–1^ and good conductivity, reaching 5.87 S m^–1^. The optimal hydrogel composition displayed a high-power factor of 4187 μW m^–1^ K^–2^, and an impressive ionic i*ZT* value of 3.5, showcasing the potential of lignin-based hydrogels for ionic thermoelectric systems. This research suggests a promising avenue for addressing environmental and economic challenges in energy production.

## Graphical abstract

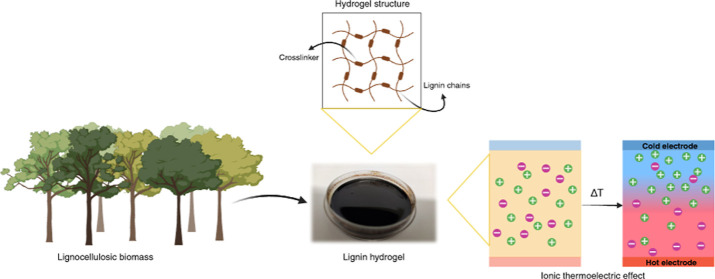

## Introduction

The demand for sustainable energy is urgent due to rising energy needs and the importance of protecting the environment. One significant problem is the considerable amount of energy wasted as heat in industrial processes, estimated to represent 20 to 50% of total energy consumption.^1^ This issue can be tackled using technologies that efficiently capture this abundant but underutilized resource, thereby enhancing overall energy production efficiency.

The Organic Rankine Cycle (ORC) and Kalina Cycle have been traditionally used to harness some of the largest waste heat sources, such as industrial waste heat and waste heat from data centers as sources of energy. These cycles utilize the heat to boil an organic working fluid (ORC) or a zeotropic mixture (Kalina Cycle) to spin a turbine in order to produce electricity, thus increasing overall energy efficiency.^2,3^ However, despite their potential, both cycles require careful calibration and maintenance to ensure optimal performance, adding operational costs.^4^ Thus, while these cycles offer promising methods for harnessing waste heat, their efficiency limitations highlight the need for continuing research and development to overcome these challenges and maximize their potential in energy recovery applications.

Searching for additional methods to address this energy imbalance, the focus has turned to thermoelectric generators (TEGs), solid-state devices designed to directly convert temperature differences into electrical energy, thereby unlocking another route to harness the potential of heat as a viable energy source.^5^ While traditional thermoelectric materials have made significant contributions to energy conversion, their sustainability and cost-effectiveness are often compromised by the reliance on rare and expensive materials such as bismuth, tellurium or selenium.^6^ Consequently, their scalability and versatility remain limited.

A useful magnitude when comparing the efficiency of different thermoelectric materials is the thermoelectric figure of merit, *ZT*, defined as the maximum efficiency of the energy conversion process at a given temperature point. *ZT* is described by the following equation

$$ZT=\frac{S^{2}\sigma T}{\kappa }$$ 1

where *S* is the Seebeck coefficient, *σ* the electrical conductivity, *κ* is the thermal conductivity with electronic (*κ*_E_) and lattice thermal (*κ*_L_) components, and *T* the absolute temperature of the thermoelectric material.^7^

Emerging as a promising alternative, ionic thermoelectric materials offer a cost-effective approach due to their utilization of polymers as the core components.^8^ In the context of ionic thermoelectric materials, the figure of merit (*ZT*) remains as important a metric for assessing efficiency as it does in traditional thermoelectric materials, but its application slightly differs.^9^ Ionic thermoelectric materials, unlike traditional electronic ones, rely on the movement of ions rather than electrons to generate electricity from heat differentials through the Soret effect. In this context, the figure of merit for ionic thermoelectric materials considers factors such as ionic conductivity, Seebeck coefficient, and thermal conductivity of the material.^10^ Unlike electronic thermoelectric materials, ionic thermoelectric materials face unique challenges in their evaluation due to their distinct conductive properties and inability to integrate directly into conventional thermoelectric generators. Therefore, in assessing the figure of merit for ionic thermoelectric materials, researchers often focus on optimizing ionic conductivity and maximizing the Seebeck coefficient while minimizing thermal conductivity to enhance overall efficiency in converting heat into electricity.^11,12^

In recent years, significant advancements have been made in the development of ionic thermoelectric materials and devices, particularly those based on inorganic chalcogenides, demonstrating their potential for energy harvesting applications. For example, Ag_2_S_0.55_Se_0.45_-based and Cu_2_Se-based systems have shown promising thermoelectric properties, including high *ZT* values and the ability to effectively recover low-grade waste heat.^13,14^ These materials, while effective, are often hindered by challenges such as reliance on rare elements, limited flexibility, and the environmental impacts associated with their synthesis and disposal.^15–17^ By addressing these limitations, polymer-based ionic thermoelectric materials, such as those utilizing lignin, offer a sustainable and cost-effective alternative. The use of renewable polymers not only mitigates environmental concerns but also broadens the applicability of ionic thermoelectric materials, paving the way for their integration into a wider range of energy harvesting applications.

The predominant use of synthetic polymers in the production of polymeric ionic thermoelectric materials offers several challenges.^18^ Synthetic polymers often rely on nonrenewable resources and can have detrimental environmental impacts during production and disposal.^19^ Additionally, their fabrication processes may involve the use of toxic chemicals and generate high levels of waste. These issues represent a problem in terms of sustainability. Here, we propose the use of a biopolymer, lignin, as an alternative. Lignin is a natural, renewable polymer derived from plant biomass, making it more sustainable and environmentally friendly, which is normally treated as a waste product of the pulp and paper industry.^20^ Thus, we can reduce dependence on fossil fuels and mitigate the environmental footprint associated with polymer production.^21^ Furthermore, lignin-based materials may exhibit unique properties that enhance their performance in thermoelectric applications, in this case, to produce hydrogels. The presence of charged functional groups within lignin facilitates the production of hydrogels with tunable properties, enhancing their versatility and utility in various fields.^22,23^ Moreover, the innate structure of lignin enables effective control over swelling behavior, crucial for optimizing hydrogel performance in diverse environments, potentially leading to improved efficiency and effectiveness in energy conversion. Thus, lignin-based hydrogels not only promote sustainability but also provides opportunities for fine-tuning their properties to meet specific functional requirements.^24^

In this study, hydrogels were produced using lignin cross-linked with poly­(ethylene glycol) diglycidyl ether (PEGDGE) and potassium hydroxide (KOH) solutions as ion providers. Their thermoelectric properties were characterized to investigate the potential use of lignin as a base for sustainable ionic thermoelectric materials. The hydrogels were synthesized by dissolving lignin in KOH solutions of different concentrations and subsequently cross-linking them with different ratios of PEGDGE. Ions from KOH act as charge carriers, producing voltage via the Soret effect under a temperature gradient.

## Experimental Section

### Materials

Alcell organosolv hardwood lignin (TCA, Tecnaro GMbH, Ilsfeld, Germany) with a weight-average molar mass (*M*_w_) of 4000 g/mol was used in this study. Potassium hydroxide (KOH) pellets of purity greater than or equal to 85% and poly­(ethylene glycol) diglycidyl ether (PEGDGE) with a molar mass of 500 g/mol were purchased from Sigma-Aldrich (St. Louis, MI, United States).

### Hydrogel Synthesis

Lignin-based hydrogels were prepared by changing two variables: the concentration of KOH used in their production (2.0, 3.3, 4.0 and 6.0 M) as well as the lignin-cross-linker mass ratio (1/0.5, 1/0.75 and/1/1). Accordingly, a total of 12 hydrogel samples were produced (see Table S1).

The lignin-based hydrogels were prepared by addition of KOH into 10 mL of deionized (DI) water and subsequent stirring at room temperature to prepare a KOH solution of the desired concentration. For the next step, 4 g of organosolv lignin were added into the solution and stirred for 4 h at 60 °C to ensure complete dissolution of the lignin. After cooling to room temperature, PEGDGE cross-linker was added dropwise to the solution until the desired lignin/PEGDGE ratio was achieved and stirred for 30 min at room temperature. The mixture was cast into round molds and left overnight at room temperature to complete the cross-linking process. This process for the preparation of lignin-derived ionic thermoelectric hydrogels is shown in Figure 1a. The mechanism for the cross-linking of the hydrogels is depicted in Figure 1b. Cross-linking occurs through a nucleophilic ring-opening reaction between the PEGDGE and hydroxide ions in lignin, which is promoted by the basic media.

**Figure 1:**
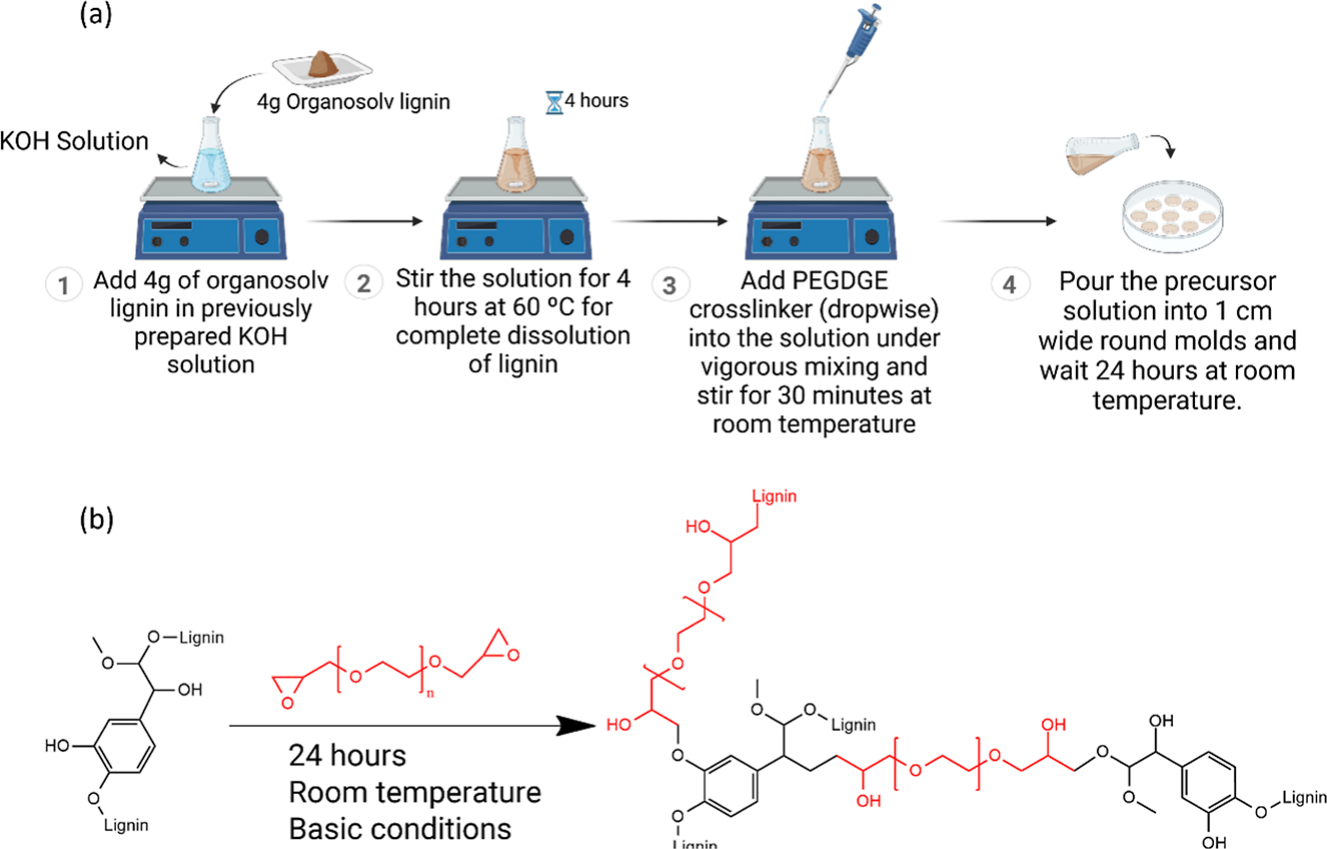
(a) Diagram of lignin-derived ionic thermoelectric hydrogels preparation. (b) PEGDGE-mediated lignin chemical cross-linking mechanism.

Finally, the hydrogels were sealed between two stainless steel electrodes with the aid of epoxy resin to facilitate the determination of the thermoelectric properties, and additionally, to slow down degradation of the sample.

### Characterization

The morphology of the lignin-based hydrogels was studied by scanning electron microscopy (SEM) using a Hitachi SU-4800 (Hitachi High-Technologies Corporation, Tokyo, Japan) and field emission scanning electron microscopy (FESEM) using a SCIOS 2 FIB-SEM (Thermo Fisher Scientific, Massachusetts, US). The samples were metallized with a gold/palladium film and the measurements were done with an acceleration voltage of 3.00 kV and a current of 1.00 nA.

FTIR was performed using an Agilent Cary 630 FTIR spectrophotometer (Agilent Technologies, California, US) using transmittance mode, in the range, 500–4000 cm^–1^.

Swelling tests of all the hydrogel samples were conducted at room temperature in water and KOH electrolyte. The swelling percentage of the gels was calculated as follows

2 $$\%\;swelling=\frac{W_{s}-W_{d}}{W_{d}}\times 100$$

where *w*_s_ is the weight of the sample at a given time, and *w*_d_ is the dry weight of the sample.

Rheology measurements were carried out in a Kinexus Prime lab+ rheometer (NETZSCH Analyzing & Testing, Selb, Germany) operating at a single frequency (1 Hz) and maintaining the temperature constant at 25 °C. A 1% shear strain was applied for 24 h per sample to measure the changes in viscoelastic properties over time.

### Thermoelectric Characterization

The Seebeck coefficient (*S*) of the hydrogels was determined with a custom-made setup. The hydrogels samples were positioned between two Peltier cells coupled with two copper blocks connected to an Agilent 34401A voltmeter. The temperature difference induced in the sample by the Peltier cells was determined through infrared (IR) imaging using an Optris Xi 400 thermographic camera (Optris, Berlin, Germany). The Seebeck coefficient was determined through a representation of open-circuit voltage (*V*) versus temperature difference (Δ*T*) over time, with S being the slope of the trendline. The values of the plot, open-circuit voltage (*V*) versus temperature difference (Δ*T*), were obtained once voltage is stable for a given temperature difference. The measurements were conducted in the out-of-plane direction.

The ionic resistance (*R*) of the hydrogels was calculated by electrochemical impedance spectroscopy (EIS) by applying 10 mV AC and sweeping the frequency from 100 kHz to 1 Hz. The corresponding *R* is the value where the impedance response intersects the *x*-axis. The ionic conductivity (σ_i_) was obtained with the following formula

3 $$\sigma_{i}=\frac{d}{A\times R}$$

where *A* is the contact area between the sample and the electrodes and *d* is the sample thickness.

A custom-made setup was built to measure the thermal conductivity of the samples. Two surface self-adhering thermocouples (Omega Engineering, Connecticut, US) were used to measure the temperature difference between the hot and cold sides. A FHF05 (Hukseflux Thermal Sensors BV, Delft, Netherlands) heat flux sensor with a sensitivity of 0.57 μV W^–1^ m^2^ was used to measure the heat flux through the sample. The heat flux was monitored by the voltage changes detected by the sensor and recorded by the 34420A multimeter (Keysight Technologies, California, US), while the heat flux sensor measured the temperature. The hot part was kept at a constant temperature (308 K) using an Omega model CN7500 temperature controller connected to the Peltier TEC1-12706 and a Pt100 temperature sensor. Besides, for Sink (cold part), a Peltier ATS-TEC30-36-017 was used. The heat flux (*Q*) was determined according to the following equation specific for the sensor

4 $$Q=\frac{V}{S\times \left(1+0.002\left(T_{Abs}-20\right)\right)}$$

where *V* is the output voltage obtained by the heat flux sensor, *s* is the sensor sensibility and *T* the absolute temperature. The thermal conductivity (*κ*) of the samples was calculated from the following equation

5 $$Q=-\kappa \frac{\Delta T}{\Delta x}$$

where *Q* was the previously obtained heat flux value, Δ*T* was the temperature difference across the sample, and Δ*x* was the distance of heat transfer (the thickness of the sample).^25^

## Results and Discussion

The objective of this study was to determine the thermoelectric performance of the 12 produced hydrogel samples. Additionally, different characterization techniques were employed to explain the changes that KOH concentration and cross-linker ratio induced in the hydrogels. The resultant material demonstrated potential for low-grade heat harvesting, an important property given that such sources comprise a significant portion of global waste heat.^26^

The selection of KOH as the electrolyte was based on prior experience of the group in electrolyte infiltration techniques applied to lignin hydrogels, coupled with the favorable solubility characteristics of organosolv lignin in alkaline solutions.^27^ Past successful experiences with KOH electrolytes in achieving effective ion transport within lignin-based systems, highlighted its suitability for facilitating ion migration and enhancing the overall performance of the thermoelectric material. Furthermore, the basic media promotes the cross-linking reaction given that it includes an epoxy ring-opening step (Figure 1b) by providing abundant hydroxide ions, enhancing nucleophilic attack on epoxy groups. Additionally, alkaline conditions provide a media for deprotonation of hydroxyl groups, enhancing their reactivity. The resulting hydrogels possessed a strong mechanical stability, being resistant to simple cutting, crushing, and stretching stress tests. The appearance was that of a soft, black-colored gel, with increasing toughness as cross-linker ratio increases.

Morphological study of the samples was performed through scanning electron microscopy (SEM) on cross sections of the hydrogels with the three different cross-linker concentrations. The results, presented in Figure 2a–c, confirm the existence of a porous hydrogel structure with channels through which the charge carrying ions can diffuse freely. The pore size and pore density in the hydrogels varies significantly with cross-linker concentration as demonstrated in the differences between Figure 2a–c. Similar results are observed in hydrogels of other KOH concentrations as can be found in the Supporting Information (Figure S1). This observation can be explained by the cross-linker being the main contributor to the formation of the cross-linked structure, thus affecting the physical properties with its concentration. Additionally, the likely presence of hydrogen bonding has secondary effects on these properties. The results indicate that a higher cross-linker ratio results in a more compact and dense structure, with reduced porosity and pore size.^28^

**Figure 2:**
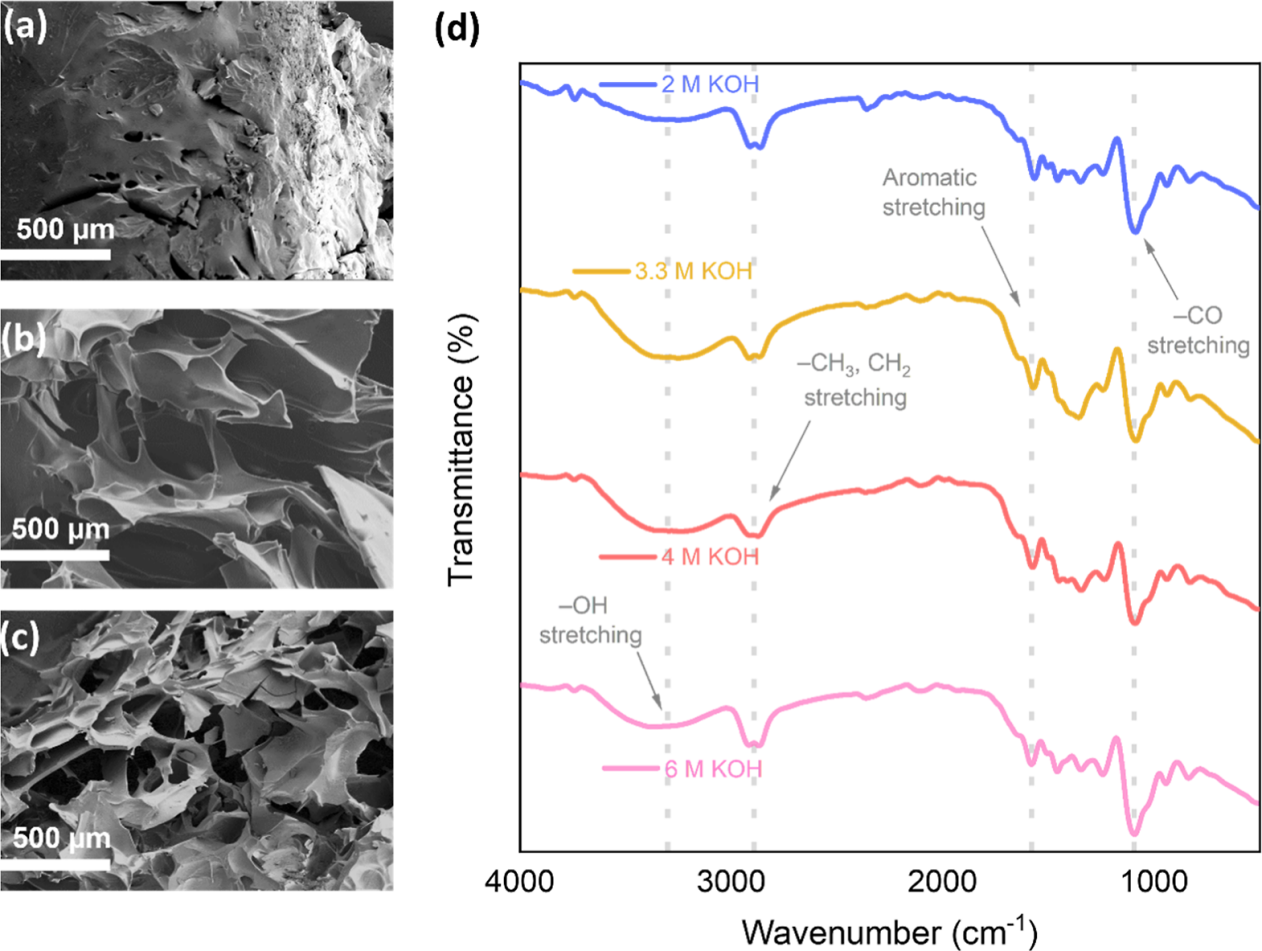
Cross-section SEM images of 2.0 M KOH lignin-derived hydrogels with different lignin/cross-linker mass ratios (a) 1/1 (b) 1/0.75 (c) 1/0.5 and (d) FTIR spectra for hydrogels with 1/0.75 lignin/cross-linker mass ratios and different KOH concentrations.

Figure 2d shows the FTIR spectra of hydrogels prepared using different KOH concentrations, enabling an examination of the influence of KOH concentration on the chemical composition of the hydrogels. The bands below 800 cm^–1^ are attributed to the aromatic C–H bonds within the lignin structure and there is no apparent difference among the spectra. The peak at 1090 cm^–1^ is attributed to C–O vibrations in ether groups, which come primarily from the etheric bonds formed by the cross-linker as previously discussed in Figure 1b. The increase in intensity of these bands is attributed to a more efficient cross-linking in more alkaline medium. Primary aliphatic alcohols and phenolic alcohols, which are also present in lignin, are also included within this peak as evidenced by the secondary bands at slightly lower wavenumbers (∼1060 cm^–1^), particularly noticeable in the spectrum of the 2.0 and 3.3 M samples.^29^ The bands observed in the 1400–1600 cm^–1^ region are related with the presence of aromatic C=C bonds, highly abundant in lignin. The peak at 2890 cm^–1^ corresponds to the stretching vibrations of C–H bonds in aliphatic and aromatic methyl groups, these groups do not interact with either the cross-linker or the OH^–^ ions, and no significant variation is observed among the different spectra.^30^ A broad band within the 3200–3400 cm^–1^ range corresponds to the stretching vibrations of O–H bonds, the increase in intensity of the band indicates an increase in the presence of aliphatic alcohols in the cross-linked samples.^31^ These alcohols are a result of the ring-opening reaction that occurs during cross-linking (Figure 1b), further supporting the idea that cross-linking is more efficient in a more alkaline environment. The findings from these FTIR spectra imply that elevating the KOH concentration results in an increased level of cross-linking within the hydrogels, thus contributing to the formation of a more stable and compact polymer network. The rest of the spectra are shown in Figure S2.

A rheological study of the hydrogels was performed by measuring changes in viscoelastic properties over time, starting from the moment the cross-linker is added. The purpose of these tests was to assess the impact of cross-linker ratio and KOH concentration on both the gelation time and the physical properties of the hydrogels. Gelation time, a critical parameter in the hydrogel formation process, was examined to detect any correlations with cross-linker concentrations. To do this, the gelation time was taken as the point in time when the initially lower elastic modulus (*G*′) and the viscous modulus (*G*″) meet. Thus, one focus of the study was on the transition from a solution to a gel state, assessing how alterations in cross-linker concentrations influenced the temporal aspect of this process. Additionally, the physical properties of the resulting hydrogels, such as the elastic modulus at the end of the cross-linking process, were used as an indicator of the mechanical strength of the hydrogels.

Figure 3 shows the dependence of *G*′ and *G*″ on the reaction time, as well as on the cross-linker ratio. Comparative analysis across samples with varying cross-linker concentrations indicates that an augmentation in cross-linker concentration corresponds to accelerated gelation times. Specifically, ∼6 h gelation time for the lowest cross-linker concentration (Figure 3a), ∼4 h gelation time for the intermediate cross-linker concentration (Figure 3b) and ∼2 h gelation time for the highest cross-linker concentration (Figure 3c). Additionally, Figure 3d shows that there is an approximately lineal relation between cross-linker ratio and gelation time for the studied samples. In addition, *G*′ clearly increases a function of the cross-linker ratio evidenced that a more compacted structure is formed which improves the ability of the material to store mechanical energy.

**Figure 3:**
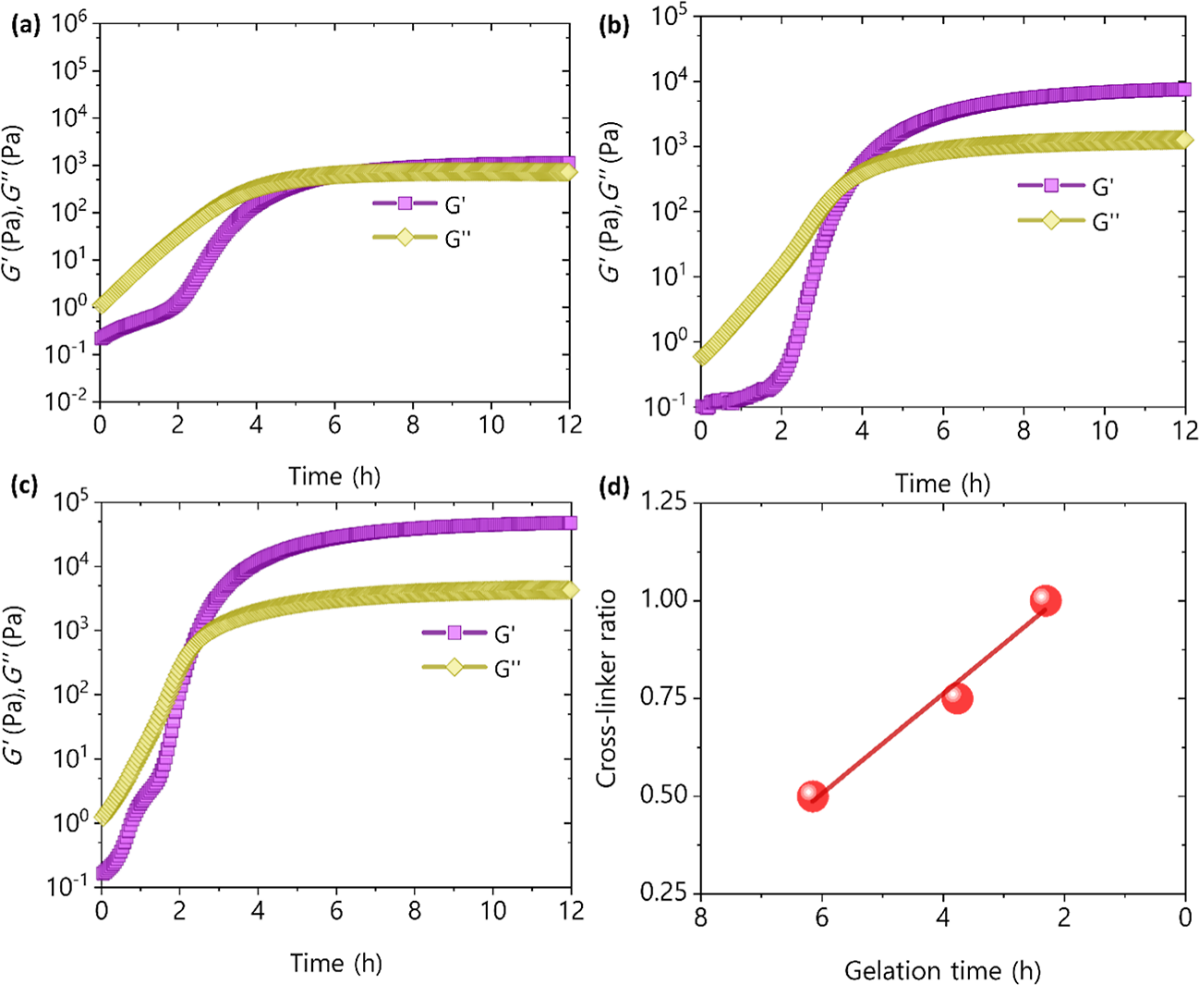
Elastic modulus and viscous modulus versus time for 3.3 M KOH samples and different lignin/cross-linker mass ratios (a) 1/0.5 (b) 1/0.75 and (c) 1/1. (d) Gelation time as a function of lignin/cross-linker mass ratio.

Similar results are observed in Figure 4, which shows the dependence of *G*′ and *G*″ on the reaction time, as well as on KOH concentration. Here, a trend of acceleration of the cross-linking with increasing KOH concentrations is observed, resulting in lower gelation times. Specifically, ∼4.3 h gelation time for 2 M KOH (Figure 4a), ∼3.8 h gelation time for 3.3 M KOH (Figure 4b), ∼2.6 h gelation time for 4 M KOH (Figure 4c) and ∼1.3 h gelation time for 6 M KOH (Figure 4d). Similarly, Figure 4e shows an approximately lineal relation between KOH concentration and gelation time for the studied samples. This can be explained by the ethylene oxide ring-opening mechanism, in which the nucleophilic character of phenolic groups increases in a basic medium. This increase in nucleophilicity facilitates the kinetics of the cross-linking process between lignin and PEGDGE. In this context, this enhanced nucleophilicity promotes the opening of ethylene oxide rings on PEGDGE, leading to faster and more efficient cross-link formation. Thus, the higher OH^–^ concentration in basic conditions accelerates the cross-linking reaction, improving the kinetics of bond formation between lignin and PEGDGE and contributing to a stronger, more stable polymer network. Furthermore, these results are in line with those found in the FTIR study of the samples (Figure 2d), where it was found that elevating the KOH concentration results in an increased level of cross-linking within the hydrogels. Both the increase in cross-linker concentration and in KOH concentration is also accompanied by heightened mechanical strength after the cross-linking is over as represented through *G*′ in Figures 3a–c and 4a–d. With a significantly higher *G*′ (∼10^5^ and ∼10^6^ Pa) in Figures 3c and 4d compared to Figures 3a and 4a (∼10^3^ and ∼10^4^ Pa).

**Figure 4:**
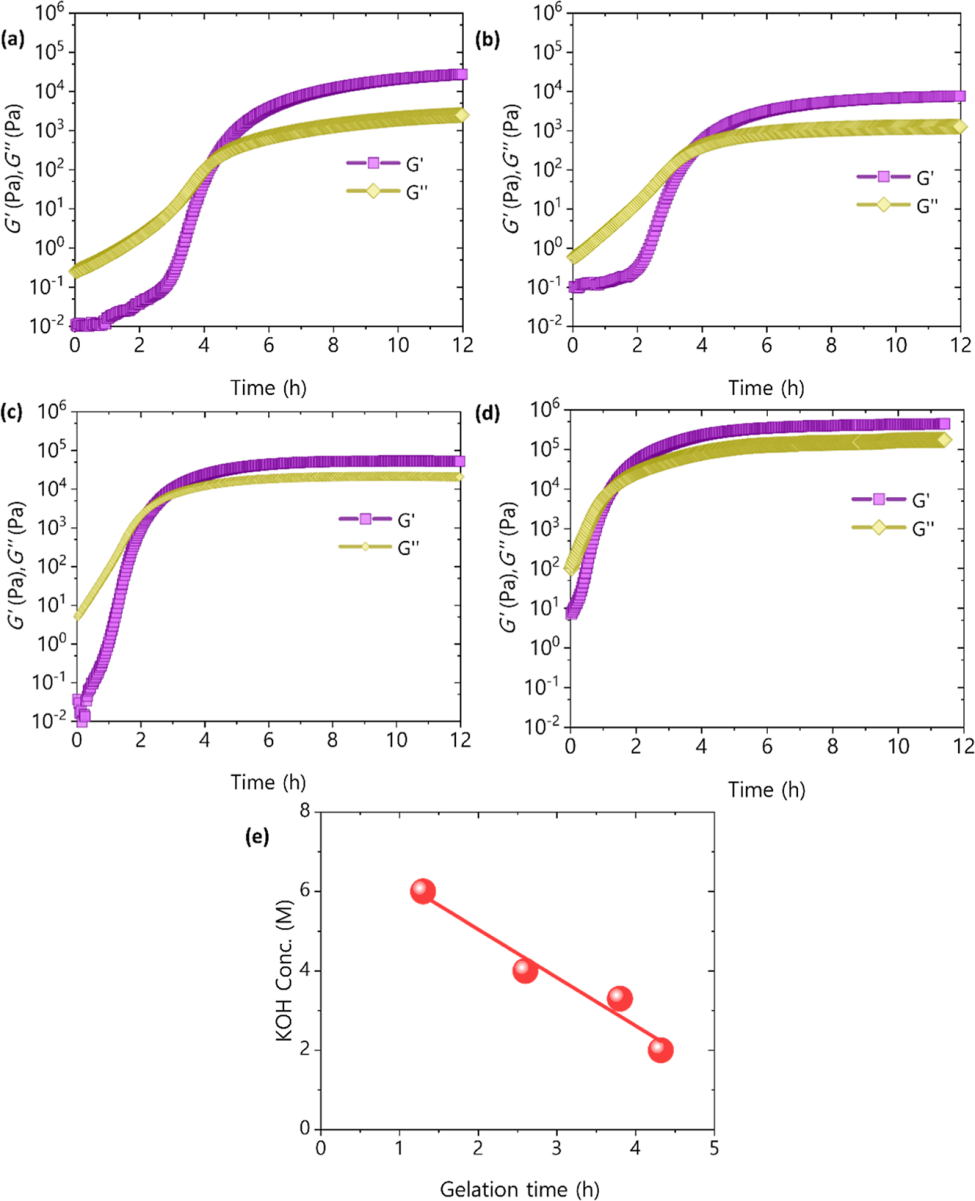
Elastic modulus and viscous modulus versus time for 1/0.75 lignin/cross-linker ratio samples and different KOH concentrations (a) 2 (b) 3.3 and (c) 4 (d) 6 M (e) gelation time as a function of KOH concentration.

The swelling capacities of the hydrogels were investigated to learn of the key factors influencing their water absorption properties and to discover their water uptake capacity, since this is important for the possible infiltration of different electrolytes. The tests were done by immersion in DI water at room temperature. Figure 5 plots swelling ratio as a function of swelling time for hydrogels under study as a function of lignin/cross-linker ratio in Figure 5a and as a function of KOH concentration in Figure 5b. Hydrogels featuring a lower cross-linker ratio exhibited a substantially higher swelling capacity, with the maximal swelling ratio observed in the hydrogel synthesized with 2 M KOH and 1/0.5 cross-linker ratio at a value of 118% depicted in Figure 5a. At the same time, Figure 5b illustrates a drastic decrease in the swelling ratio as the concentration of the KOH utilized to synthesize the hydrogels increases. This can be attributed to a saturation of ions in the gel matrix diminishing its water uptake capacity. The results highlight the role that both the cross-linker ratio and the concentration of KOH play during the synthesis process of the hydrogels, resulting in varying swelling capacities for the different hydrogels. However, these findings suggest that all synthesized hydrogels possess an adequate water uptake capacity, rendering them suitable for applications involving electrolyte infiltration and i-TE applications.

**Figure 5:**
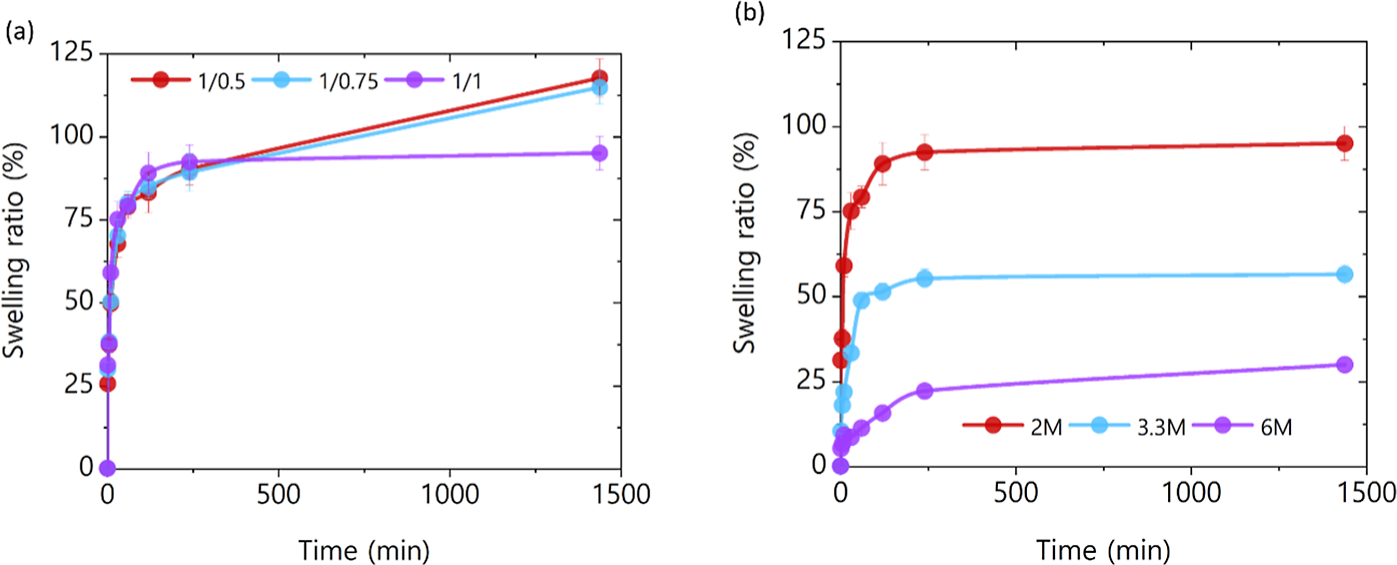
Swelling capacity in water of (a) 2.0 M KOH lignin-derived hydrogels with different lignin/cross-linker mass ratios and (b) 1/1 lignin/cross-linker mass ratio lignin-derived hydrogels with different KOH concentrations.

The investigation into the ionic-thermoelectric (i-TE) properties of lignin-derived hydrogels constituted a critical aspect of our study. This phase involved subjecting all variants of the hydrogels to i-TE testing, the resulting measurement data is represented in Figures S3–S5. The primary objective was to discern the optimal electrolyte concentration and cross-linker ratio based on the evaluations of ionic Seebeck coefficient values and ionic conductivity, as depicted in Figure 6a/b. Notably, higher KOH concentrations resulted in higher Seebeck coefficient in the hydrogels. This effect can be explained by the increased concentration of KOH, which deprotonates a greater number of hydroxyl (−OH) groups within the lignin-based cross-linked structure. This deprotonation process converts the hydroxyl groups into negatively charged phenoxide ions, thereby immobilizing negative charges within the lignin network.^32^ As a result, potassium ions (K^+^) become the primary mobile ionic species within the system. With K^+^ ions as the dominant ionic species, their migration under a thermal gradient is enhanced, contributing to an accumulation of K^+^ ions at the cold electrode.^33,34^ This accumulation results in a higher ionic Seebeck coefficient, as the thermoelectric response is driven by the concentration gradient of K^+^ ions between the hot and cold electrodes. Thus, the increased KOH concentration not only stabilizes negative charges on the lignin matrix but also improves ionic conductivity and thermoelectric performance by increasing the Seebeck coefficient through the accumulation of K^+^ ions at the cold electrode.^27^ Furthermore, the optimal cross-linking ratio of 1:0.75 demonstrates that this particular network structure is best suited to enhance the ionic Seebeck coefficient. This ratio likely represents the ideal balance between cross-link density and network flexibility, enabling efficient thermodiffusion of K^+^ ions across the material. When the cross-link density is too high, the tightly interconnected network can impede ion mobility, limiting the movement and accumulation of K^+^ ions at the cold electrode, which is crucial for achieving a high Seebeck response. In contrast, a moderately cross-linked network, as indicated by the 1:0.75 ratio, provides enough structural integrity to prevent excessive ion leakage while maintaining sufficient free volume and flexibility to facilitate the thermodiffusion process. This configuration allows K^+^ ions to migrate more effectively along the thermal gradient, leading to a more pronounced ion accumulation at the cold side, which is necessary to maximize the ionic Seebeck coefficient. Therefore, this specific cross-link ratio achieves an ideal microstructure that optimally balances ion transport pathways and network stability, promoting a stronger thermoelectric effect. The trend of ionic conductivity as a function of the cross-linking ratio is complex to explain due to multiple factors influencing conductivity. Key aspects include the free volume of water within the hydrogel network, its viscosity, and ionic interactions at high electrolyte concentrations. For typical electrolyte solutions, ionic conductivity decreases at higher concentrations due to ion pairing and association.^35,36^ At these levels, oppositely charged ions can form pairs or clusters, reducing the number of free charge carriers and thus lowering conductivity. This effect is particularly evident in samples with a cross-linking ratio of 1/0.5. The higher free volume within the hydrogel network, resulting from the lower cross-linking ratio, facilitates the formation of ionic pairs, thereby reducing ionic conductivity as the electrolyte concentration increases. However, the ionic conductivity values for the 1/0.5 ratio are lower compared to those of samples with a cross-linking ratio of 1/0.75, indicating better ion mobility in the latter. This difference can be attributed to the presence of non-cross-linked polymer chains dissolved in the free water volume of 1/0.5 hydrogels. These chains increase the viscosity of the system, impeding ion mobility within the hydrogel network. In contrast, hydrogels with cross-linking ratios of 1/0.75 and 1/1 exhibit a peak in ionic conductivity at a 3.3 M KOH concentration. This suggests that a more highly cross-linked polymer network can suppress ionic pair formation to a certain extent, thereby increasing the number of free ions. Beyond 4 M KOH, however, ionic conductivity decreases for all samples due to excessive ionic pair formation. For hydrogels prepared with a cross-linking ratio of 1/1, a similar trend is observed, but with slightly lower ionic conductivity compared to the 1/0.75 samples. This difference arises because highly cross-linked polymer networks significantly reduce the water uptake capacity of the hydrogels, thereby limiting ion mobility and, consequently, ionic conductivity. Overall, the hydrogel synthesized with 4 M KOH, and a 1/0.75 lignin/cross-linker ratio exhibited markedly superior ionic thermoelectric responses when compared to other variants. The recorded ionic Seebeck coefficients ranged from 9 ± 2 mV/K to 30 ± 6 mV/K. Additionally, the ionic conductivity values ranged from 0.30 ± 0.02 S/m and 5.9 ± 0.4 S/m. Subsequent calculation of the power factor revealed values ranging from 150.04 to 4187.94 μW/(m·K^2^) as presented in Figure 6c. Table S2 provides a comprehensive collection of all Seebeck, ionic conductivity and power factor values.

**Figure 6:**
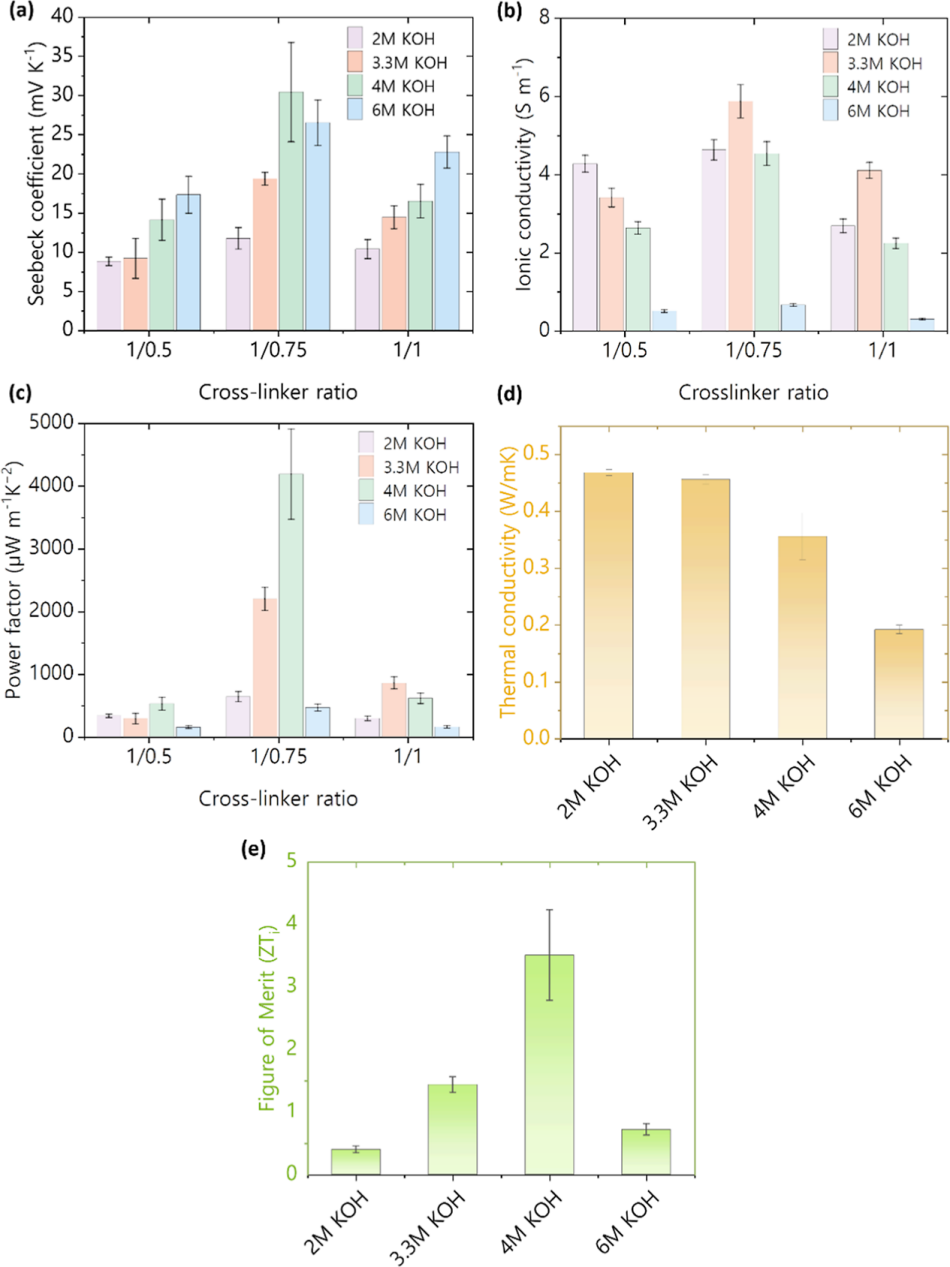
Influence of KOH concentration and cross-linker ratio on the ionic thermoelectric characteristics of lignin-derived hydrogels, evaluated in terms of (a) ionic conductivity, (b) ionic Seebeck coefficient, (c) power factor, (d) thermal conductivity and (e) figure of merit.

We can conclude that hydrogels with a 1/0.75 cross-linker ratio exhibited the most favorable thermoelectric performance, prompting their selection for further investigation. This part of the study involved assessments of thermal conductivity values at various electrolyte concentrations, as depicted in Figure 6d. The measured thermal conductivity values ranged from 0.19 to 0.47 W/mK. The highest thermal conductivity, 0.47 W/mK, was noted in the hydrogel synthesized with 2 M KOH, while the lowest, 0.19 W/mK, occurred in the hydrogel with a 6 M electrolyte. The decrease in thermal conductivity can be attributed to water uptake during the swelling process, considering the thermal conductivity of distilled water is approximately 0.6 W/mK. Swelling of hydrogels was observed to diminish with increasing KOH concentration (Figure 5), leading to reduced electrolyte uptake and subsequently lower thermal conductivity values. These thermal conductivity values tended to approach those of precursor materials, such as lignin-based materials (0.3–0.5 W/mK) and water.

The combination of the lower thermal conductivity observed in lignin-derived hydrogels infiltrated with a highly concentrated KOH electrolyte and their exceptional power factor values yields remarkable ionic figures of merit (i*ZT*). Specifically, as shown in Figure 6e,i*ZT* values reached 1.44 and 3.51 for 3.3 and 4 M KOH hydrogels, respectively, while a lower value was observed for the 6 M KOH hydrogel due to a significant decrease in ionic conductivity. Thermal conductivity and figure of merit data can be found in Table S3. In conclusion, these findings show the significant impact of cross-linker ratios and electrolyte concentrations on the thermoelectric performance of lignin-derived hydrogels. Notably, the selected hydrogel composition with a 1/0.75 cross-linker ratio demonstrated promising thermoelectric properties, warranting further exploration. The observed trends in thermal conductivity highlight the intricate relationship between hydrogel swelling, electrolyte uptake, and thermal conductivity values.

In addition, a prototype supercapacitor device was prepared utilizing the optimum developed hydrogel and electrodes based on carbon nanotubes (CNTs). The electrodes were produced based on the ref 37 using a suspension of MWCNTs, polyvinylpyrrolidone (PVP), carbon black and *N*-methyl-2-pyrrolidone (NMP) and prepared with the drop-casting technique. The CV curve, Nyquist plot, and thermal charge–discharge cycle of the prototype supercapacitor device are presented in Figure S6. The device’s CV curve’s symmetrical leaf-like shape suggests a blend of electric double layer capacitance and pseudocapacitive charge storage.^38^ Additionally, the EIS curve showed a greater inclination toward the vertical axis, signifying EDLC characteristics and efficient ion transport between the hydrogel and electrodes. With the data from the thermal charge–discharge cycle, the power density and energy density were calculated for the second stage at 1 kΩ external load and estimated to be 25.29 μW/m^2^ and 474.02 mJ/m^2^ respectively. These results are aligned with previous published studies.^27,39,40^

## Conclusions

This study intended to explore the ionic thermoelectric properties in hydrogels derived from lignin. The presence of ionizable groups on the hydrogel surface facilitated selective ionic migration, synergizing with the Soret effect and thereby enhancing both ionic conductivity and the Seebeck coefficient. Various compositions of lignin-derived hydrogels were systematically examined to optimize the thermoelectric efficiency of ionic thermoelectric (i-TE) systems. It was discerned that hydrogels characterized by higher concentrations of KOH and a cross-linker ratio of 1/0.75 (lignin/cross-linker mass) demonstrated superior thermopower compared to others. The optimized lignin-derived hydrogel, synthesized with a 4 M KOH electrolyte, displayed noteworthy characteristics including high ionic conductivity (5.87 S/m), low thermal conductivity (0.36 W/m K), and an impressive Seebeck coefficient of 30.39 mV/K, resulting in a remarkable i*ZT* value of 3.51. This study establishes the viability of employing readily available lignin for low-grade thermal energy harvesting, addressing sustainability and cost-effectiveness concerns. These findings pave the way for the development of sustainable ionic thermoelectric devices geared toward efficient low-grade energy harvesting applications.

## Supplementary Material



## Data Availability

The data supporting this article has been included as part of the Supporting Information (Figures S3–S5) and the data sets are available upon request from the authors.

## Notes

Hydrogel compositions and synthesis details. Additional materials characterization results: S.E.M, FTIR, Seebeck, EIS. Summary table of thermoelectric data (PDF)
