# Supplementary material for: Lignin-Derived Ionic Hydrogels for Thermoelectric Energy Harvesting
Source: ACS Appl Polym Mater. 2025 Mar 3;7(5):3093–102. doi: 10.1021/acsapm.4c03816 (PMC12128191; doi:10.1021/acsapm.4c03816)
Supplement: Supplementary file 1 [file ap4c03816_si_001.pdf]

Supporting information

Lignin-Derived Ionic Hydrogels for Thermoelectric Energy Harvesting

Nicolás Menéndez[1], Muhammad Muddasar[1,2], Mohammad Ali Nasiri[3], Andrés Cantarero[3], Clara M Gómez[1], Rafael Muñoz-Espí[1], Maurice N. Collins[2,4], and Mario Culebras[1]*

[1] Institute of Materials Science (ICMUV), University of Valencia, PO Box 22085, E46071 Paterna, 46980, Spain.

[2] Stokes Laboratories, School of Engineering, Bernal Institute, University of Limerick, Limerick, V94 T9PX, Ireland.

[3] Institute of Molecular Science (ICMol), University of Valencia, PO Box 22085, E46071, Paterna, 46980, Spain

[4] SFI Centre for Advanced Materials and BioEngineering Research, Dublin, D02 PN40 Ireland.

*Corresponding author.

E-mail: Mario.Culebras@uv.es

Experimental section

Synthesis of lignin-based hydrogels

The lignin-based hydrogels were prepared by addition of KOH into 10 mL of deionized (DI) water and subsequent stirring at room temperature to prepare a KOH solution of the desired concentration. For the next step, 4 grams of organosolv lignin were added into the solution and stirred for 4 hours at 60 ºC to ensure complete dissolution of the lignin. After cooling to room temperature, PEGDGE crosslinker was added dropwise to the solution until the desired lignin:PEGDGE ratio was achieved and stirred for 30 minutes at room temperature. The mixture was cast into round molds and left overnight at room temperature to complete the crosslinking process. The prepared hydrogels were denoted as xM – 1/y CL, where x indicates the concentration of KOH and y represents the ratio of %w concentration of the PEGDGE crosslinker against lignin. Table S1 summarizes the compositions of all 12 hydrogels.

Table S1: Compositions of lignin-based hydrogels

	**Lignin (g)**	**PEGDGE (g)**	**DI water (g)**	**KOH (g)**
**2M – 1/0.5**	4	2	10	1.12
**3.3M – 1/0.5**	4	2	10	1.85
**4M – 1/0.5**	4	2	10	2.24
**6M – 1/0.5**	4	2	10	3.37
**2M – 1/0.75**	4	3	10	1.12
**3.3M – 1/0.75**	4	3	10	1.85
**4M – 1/0.75**	4	3	10	2.24
**6M – 1/0.75**	4	3	10	3.37
**2M – 1/1**	4	4	10	1.12
**3.3M – 1/1**	4	4	10	1.85

**4M – 1/1**	4	4	10	2.24
**6M – 1/1**	4	4	10	3.37

Material characterizations

The morphology of the lignin-based hydrogels was studied by scanning electron microscopy (SEM) using a Hitachi SU-4800 (Hitachi High-Technologies Corporation, Tokyo, Japan) and field emission scanning electron microscopy (FESEM) using a SCIOS 2 FIB-SEM (Thermo Fisher Scientific, Massachusetts, U.S). The samples were metallized with a gold/palladium film and the measurements were done with an acceleration voltage of 3.00 kV and a current of 1.00 nA.

FTIR was performed using an Agilent Cary 630 FTIR spectrophotometer (Agilent Technologies, California, US) using transmittance mode, in the range, 500-4000 cm^{-1}.

Swelling tests of all the hydrogel samples were conducted at room temperature in water and KOH electrolyte. The swelling percentage of the gels was calculated as follows:

$$\% \text{ swelling} = \frac{W_s - W_d}{W_d} \times 100 \qquad (2)$$

where W_s is the weight of the sample at a given time, and W_d is the dry weight of the sample.

Rheology measurements were carried out in a Kinexus Prime lab+ rheometer (NETZSCH Analyzing & Testing, Selb, Germany) operating at a single frequency (1 Hz) and maintaining the temperature constant at 25 ºC. A 1% shear strain was applied for 24 hours per sample to measure the changes in viscoelastic properties over time.

Thermoelectric characterization

The Seebeck coefficient (S) of the hydrogels was determined with a custom-made setup. The hydrogels samples were positioned between two Peltier cells coupled with two copper blocks connected to an Agilent 34401A voltmeter. The temperature difference induced in the sample by the Peltier cells was determined through infrared (IR) imaging using an Optris Xi 400 thermographic camera (Optris, Berlin, Germany). The Seebeck coefficient was determined

through a representation of open-circuit voltage (V) versus temperature difference (ΔT) over time, with S being the slope of the trendline.

The ionic resistance (R) of the hydrogels was calculated by electrochemical impedance spectroscopy (EIS) by applying 10 mV AC and sweeping the frequency from 100 kHz to 1 Hz. The corresponding R is the value where the impedance response intersects the x-axis. The ionic conductivity (σ_i) was obtained with the following formula:

$$\sigma_i = \frac{d}{A \cdot R} \qquad (3)$$

Where A is the contact area between the sample and the electrodes and d is the sample thickness.

A custom-made setup was built to measure the thermal conductivity of the samples. Two surface self-adhering thermocouples (Omega Engineering, Connecticut, US) were used to measure the temperature difference between the hot and cold sides. A FHF05 (Hukseflux Thermal Sensors BV, Delft, Netherlands) heat flux sensor with a sensitivity of 0.57 $\mu V\ W^{-1}\ m^2$ was used to measure the heat flux through the sample. The heat flux was monitored by the voltage changes detected by the sensor and recorded by the 34420A multimeter (Keysight Technologies, California, US), while the heat flux sensor measured the temperature. The hot part was kept at a constant temperature (308 K) using an omega model CN7500 temperature controller connected to the Peltier TEC1-12706 and a Pt100 temperature sensor. Besides, for Sink (cold part), a Peltier ATS-TEC30-36-017 was used. The heat flux (Q) was determined according to the following equation specific to the sensor:

$$Q = \frac{V}{S \times (1 + 0.002(T_{Abs} - 20)} \qquad (4)$$

where V is the output voltage obtained by the heat flux sensor, s is the sensor sensibility and T the absolute temperature. The thermal conductivity (κ) of the samples was calculated from the following equation:

$$Q = -\kappa \frac{\Delta T}{\Delta x} \qquad (5)$$

where Q was the previously obtained heat flux value, ΔT was the temperature difference across the sample, and Δx was the distance of heat transfer (the thickness of the sample). The

prototype ionic thermoelectric supercapacitor underwent electrochemical characterization by means of CV and EIS. CV was performed with a scanrate of 10 mV/sec along a 0-0.2V voltage rage. EIS was performed applying 10 mV AC and sweeping the frequency from 100 kHz to 10 mHz. The current density (I.D), power density (P.D), and energy density (E. D) were calculated using the following equations.

$$I.D = \frac{\mathrm{V}}{\mathrm{RA}} \tag{6}$$

$$P.D = I.D^2 \times R \tag{7}$$

$$E.D = \int \frac{\mathrm{P.D}}{\mathrm{dt}} \tag{8}$$

Where:

V = Voltage (V)

R = resistance (external load) (Ω)

A = area (m^2)

Additionally, a thermal charge-discharge cycle was induced in the device with our custom Seebeck setup, all these characterizations can be found in Figure S6.

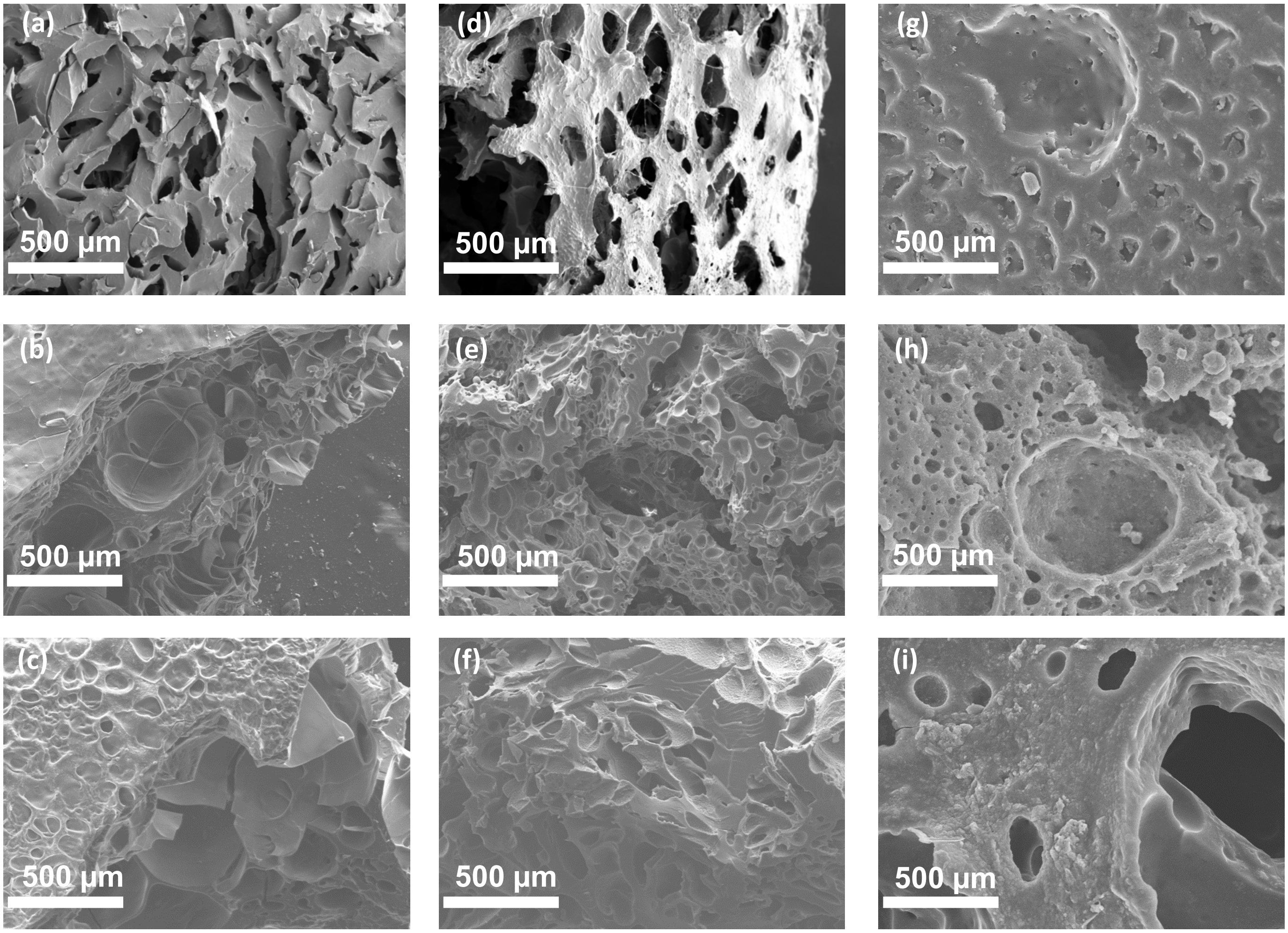

Figure S1: Cross-section SEM images of lignin-derived hydrogels with different lignin/crosslinker mass ratios and KOH concentrations. **(a)** 3.3M – 1/1 **(b)** 3.3M – 1/0.75 **(c)** 3.3M – 1/0.5 **(d)** 4M – 1/1 **(e)** 4M – 1/0.75 **(f)** 4M – 1/0.5 **(g)** 6M – 1/1 **(h)** 6M – 1/0.75 **(i)** 6M – 1/0.5

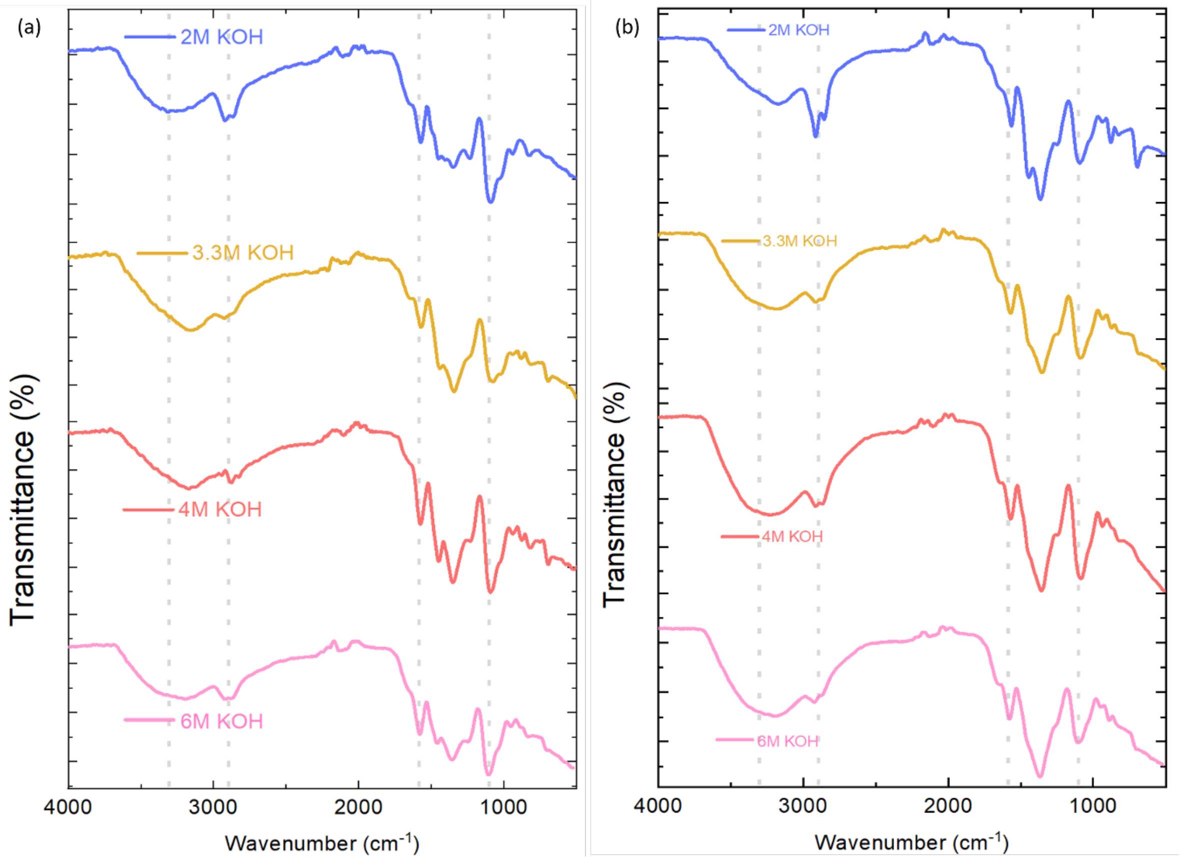

Figure S2: FTIR spectra for hydrogels with **(a)** 1/0.5 crosslinker ratio and **(b)** 1/1 crosslinker ratio.

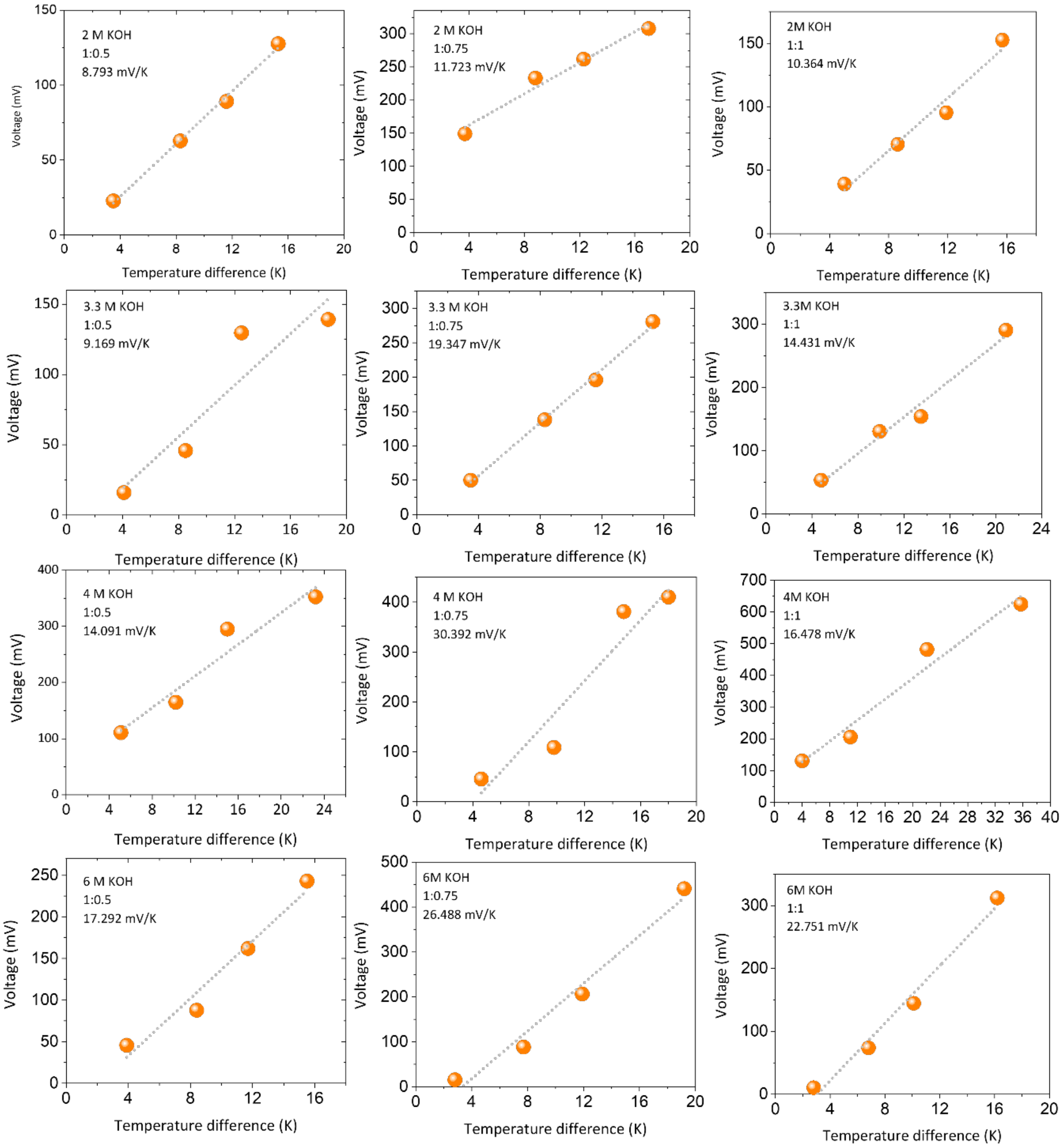

Figure S3: linear fit of the open circuit voltage vs. temperature difference for all hydrogel samples.

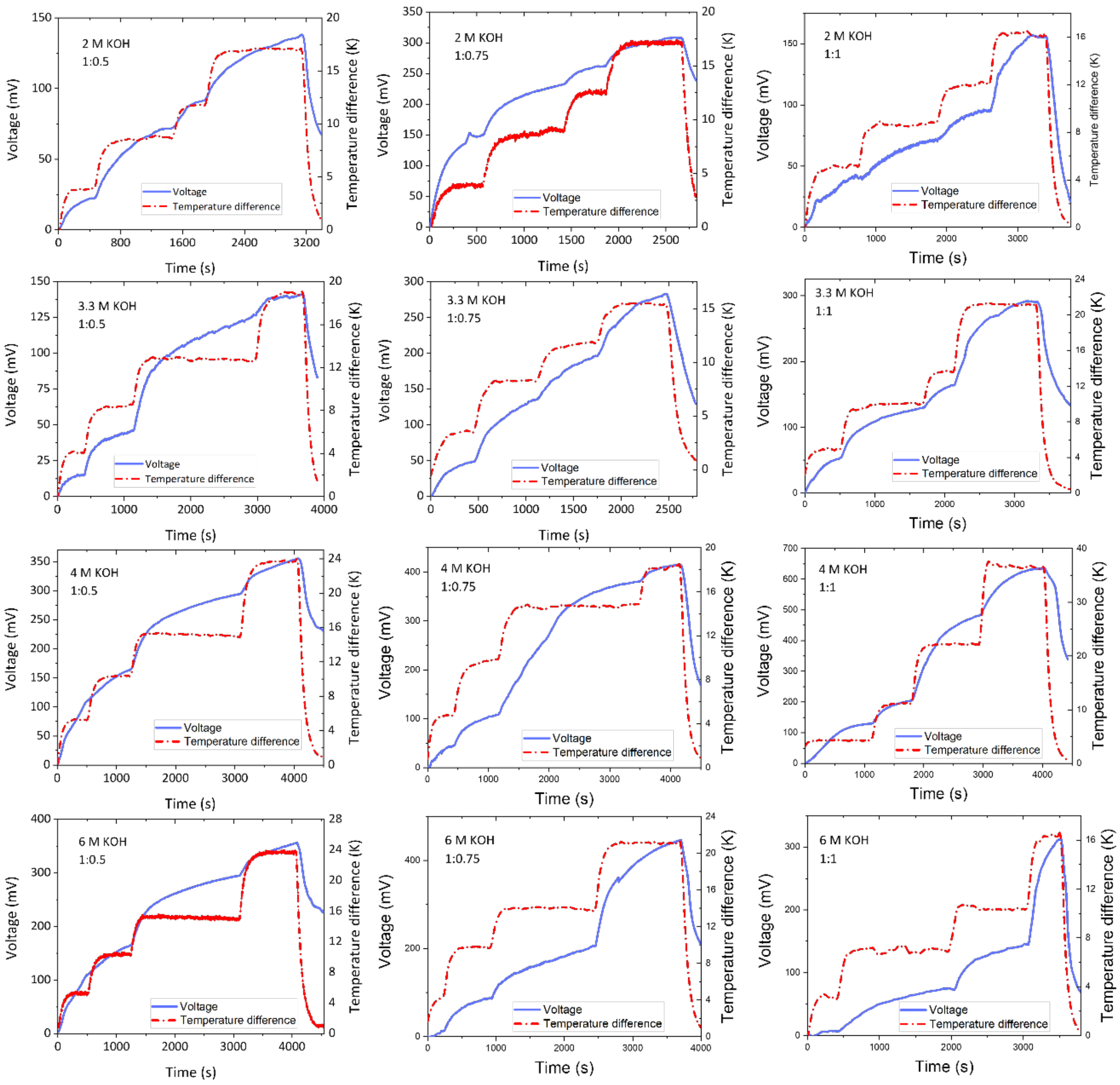

Figure S4: Voltage and temperature profile of all hydrogel samples.

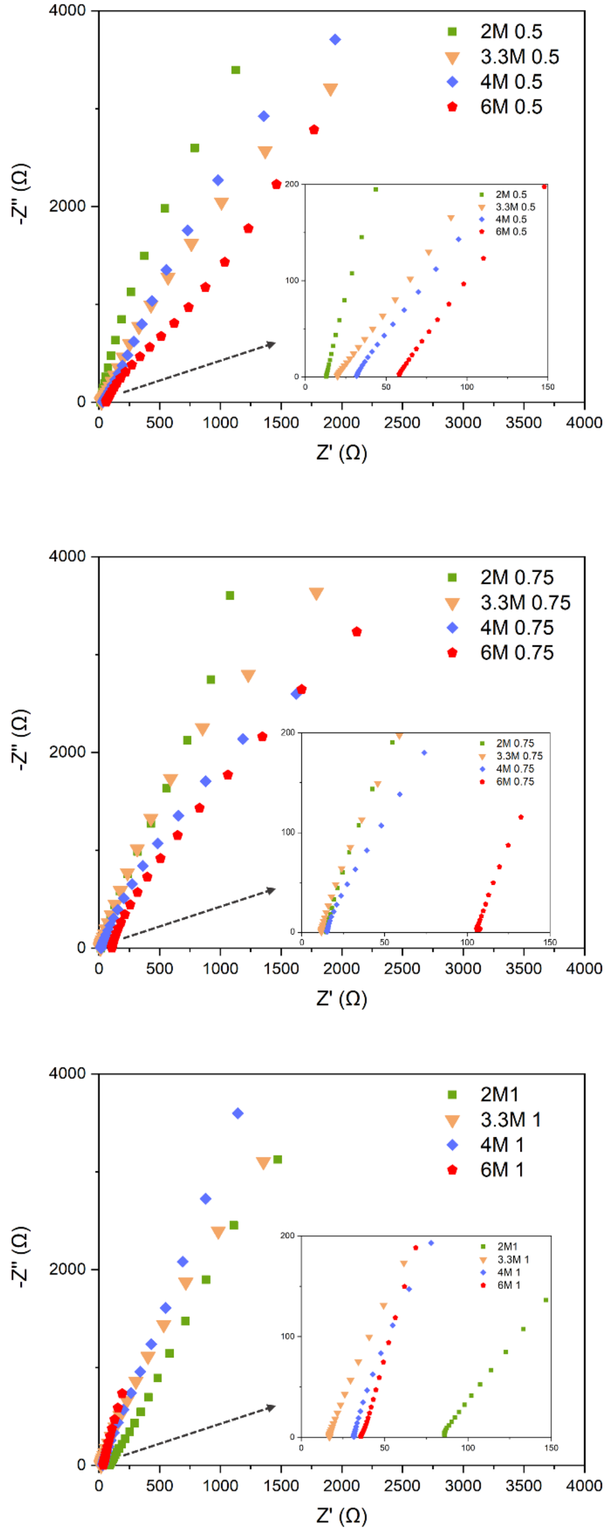

Figure S5: Nyquist plot of all hydrogels grouped by the lignin:crosslinker ratio.

Table S2: Ionic thermoelectric properties of all lignin hydrogels.

Sample	Seebeck coefficient (mV/K)	S.D (±)	Ionic conductivity (S/cm)	S.D (±)	Power factor (μW/mK²)	S.D (±)
**2M – 1/0.5**	8.793	0.533	4.273	0.214	330.375	25.970
**3.3M – 1/0.5**	9.169	2.552	3.411	0.239	286.752	82.300
**4M – 1/0.5**	14.091	2.629	2.636	0.158	523.394	102.567
**6M – 1/0.5**	17.292	2.344	0.5018	0.0356	150.045	22.956
**2M – 1/0.75**	11.723	1.375	4.625	0.259	635.608	82.430
**3.3M – 1/0.75**	19.347	0.814	5.872	0.423	2197.927	183.360
**4M – 1/0.75**	30.392	6.345	4.534	0.304	4187.936	718.308
**6M – 1/0.75**	26.488	2.917	0.6605	0.0357	463.416	56.849
**2M – 1/1**	10.364	1.224	2.687	0.175	288.617	38.920
**3.3M – 1/1**	14.431	1.454	4.105	0.205	854.882	96.134
**4M – 1/1**	16.478	2.116	2.242	0.134	608.758	86.225
**6M – 1/1**	22.751	2.045	0.2984	0.0185	154.454	16.865

Table S3: Thermal conductivity and Figure of Merit (ZT) for all hydrogels with a 1/0.75 lignin:crosslinker ratio.

Sample	Thermal conductivity (W/mK)	S.D (±)	Figure of Merit	S.D (±)
**2M – 1/0.75**	0.468	0.006	0.405	0.053
**3.3M – 1/0.75**	0.456	0.008	1.437	0.122
**4M – 1/0.75**	0.356	0.041	3.507	0.725
**6M – 1/0.75**	0.192	0.008	0.720	0.093

Figure S6c shows the thermoelectric charging profile. (i) In the first stage, cations and anions migrate toward opposite electrodes under an applied temperature gradient (~6 K), leading to a gradual voltage increase during the charging process until saturation (~38 mV). The observed reduction in the Seebeck coefficient is attributed to the high charge transfer resistance (Rct). (ii) When an external load (1 kΩ) is connected to the ionic-thermoelectric supercapacitor, electrons flow through the circuit to balance the accumulated charge, effectively charging both electrodes. (iii) As the temperature gradient is removed and the external circuit is disconnected, the ions diffuse back to their original states. However, the electrons remain at the electrodes, resulting in a negative voltage. (iv) Finally, when the external circuit is reconnected, the accumulated electrons on the electrodes flow through the load once again, but this time in the opposite direction. This phenomenon is referred to as reverse electron working or the discharging cycle.

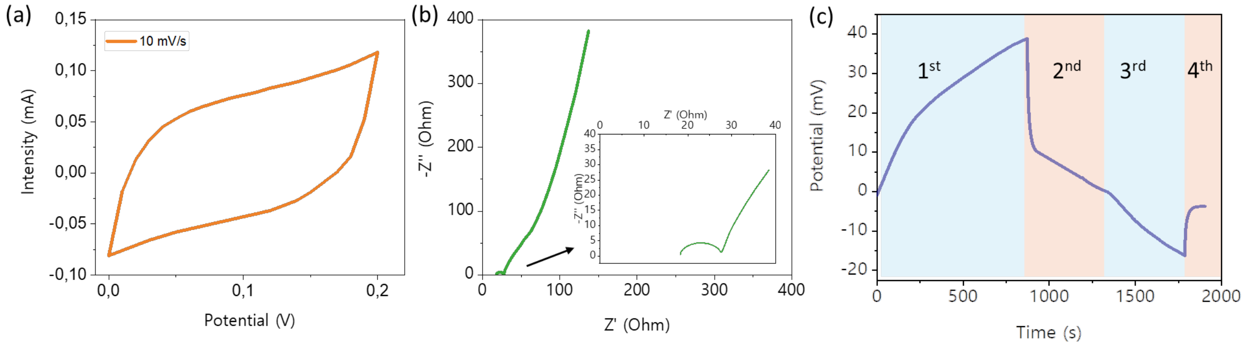

Figure S6: Characterization of ionic thermoelectric supercapacitor device. (a) Cyclic voltammetry. (b) Nyquist plot. (c) Thermal charge-discharge cycle at $\Delta T = 6$ K
